# Comparison of Simple Models of Periodic Protocols for Combined Anticancer Therapy

**DOI:** 10.1155/2013/567213

**Published:** 2013-04-07

**Authors:** Marzena Dołbniak, Andrzej Świerniak

**Affiliations:** Department of Automatic Control, Silesian University of Technology, ul. Akademicka 2A, 44-100 Gliwice, Poland

## Abstract

Several simple ordinary differential equation (ODE) models of tumor growth taking into account the development of its vascular network are discussed. Different biological aspects are considered from the simplest model of Hahnfeldt et al. proposed in 1999 to a model which includes drug resistance of cancer cells to chemotherapy. Some of these models can be used in clinical oncology to optimize antiangiogenic and cytostatic drugs delivery so as to ensure maximum efficacy. Simple models of continuous and periodic protocols of combined therapy are implemented. Discussion on the dynamics of the models and their complexity is presented.

## 1. Introduction

In the last decades, cancer became one of the most important morbidity and mortality causes. The reasons for the increasing cases of this disease vary depending on different cancer types [1]. Physical inactivity, obesity, use of postmenopausal hormone therapy or oral contraceptives, and alcohol consumption are the main risk factors for breast cancer. Colon cancer can be caused by changes in dietary patterns, obesity, and an increased prevalence of smoking. The relatively high burden of lung cancer can be a result of smoking and exposure to occupational and environmental carcinogens such as asbestos, arsenic, radon, and polycyclic aromatic hydrocarbons. The global cancer morbidity continues to increase rapidly, based on estimation described in [2]; the number of new cancer cases will rise from 12.7 millions in 2008 [1] to 21.4 millions by 2030. To this day no single effective drug for cancer has been discovered. New potential treatments are targeted therapies, and there exists a broad family of molecularly targeted anticancer drugs, one of which is antiangiogenic therapy. 

Tumor angiogenesis (blood vessel formation from existing vascular network) is one of the hallmarks of cancer [3]. Blood vessels deliver nutrients and oxygen. The idea of antiangiogenic therapy is that a tumor cannot grow beyond certain dimensions without developing its own network of blood and lymphatic vessels [4].

In [5], the gap between preclinical (mouse models—localized primary tumor) and clinical testing (late-stage metastatic) is discussed. Antiangiogenic agents are not efficient at the level suggested by clinical trials, and depending on the disease stage different results were obtained. Hundreds of clinical trials included mostly an inhibitor targeting the vascular endothelial growth factor (VEGF) pathways (one of the proangiogenic proteins). In some cases, metastatic disease progression slowed, leading to progression-free survival and overall survival benefits compared with the control, but it was not associated with survival improvements.

There is a big debate about the effectiveness of these drugs, in particular, that two types of resistance have been observed. The first, termed evasive, includes revascularization as a result of upregulation of alternative proangiogenic signals, protection of the tumor, increased metastatis, and the second, intrinsic, and includes rapid adaptive responses, in the case of pre-existing conditions defined by the absence of any beneficial effect of antiangiogenic agents [6].

Biologists suggest that antiangiogenic therapy might become an essential component of multidrug cancer therapy [7, 8], especially with chemotherapy, using angiogenic inhibitors to normalize the abnormal vasculature thereby facilitating drug delivery [9]. Some results from clinical studies of combination therapy are shown in [10]. A smaller dose of antiangiogenic agents (bevacizumab 5 mg/kg) shows significantly different (higher) median survival than chemotherapy alone in the treatment group, while a dose of 10 mg/kg can even increase survival compared to chemotherapy alone in the treatment group. Several clinical trials of combined therapy have been made recently, and some example are presented in Table 1 [11].

Continuous and periodic therapy is analyzed. The continuous treatment with angiogenic inhibitors ultimately leads to a decrease in tumor blood flow and a decreased tumor uptake of coadministrated cytotoxic drugs. In periodic therapy, the main goal of antiangiogenic agents is to normalize tumor vasculature, which may facilitate tumor cell recovery from cytostatic agents [10]. 

## 2. Models of Cancer Growth including Vascularization

There is a delicate balance between reliability and realism during building any mathematical model. In the literature, we can find several types of mathematical models used in tumor and angiogenic development. Most popular models have the form of partial differential equations (PDEs) [14, 15]. Other models were constructed using stochastic differential equations [16, 17], random walk models [18], cellular automata [19, 20], multiscale phase-field models [21], and computer algorithms describing the process of vessel formation and maturation [22]. PDEs represent the most detailed methods, including tumor localization, its geometry, and environment. Nevertheless, such models are difficult to tread by tools of mathematical analysis. Some mechanisms of cancer still remain a mystery. Each of the characteristics of tumor growth and vascularisation should be included; however, for our research we preferred to start with simple ODEs models and then to include more complex, medically significant features. 

We are aware that there is a big gap between the simulated and the real world and this is why we try to focus on several questions. The first is how modification of the basic model improves the fit between the simulated therapy protocol and the real clinical results. The second question is how the dynamics of this model will look like after implementing protocols already used in medicine.

Hahnfeldt et al. in 1999 [12] proposed a model based on experimental data from antiangiogenic therapy trials of Lewis lung tumors in mice. The main goal of this model was providing time-dependent carrying capacity for cancer under angiogenic control, being minimally parameterized, being important during application of protocols in real life, and recognizing the distinct kinetics of angiogenic stimulations and inhibitions. Two ordinary differential equations describe tumor and vascular interaction. The first shows dynamics of the tumor growth and can be expressed by a Gompertz-type equation or a logistic type. The growth in this model is bounded by the carrying capacity, which is vessel volume. In the original Hahnfeldt et al. model, a Gompertz-type ODE was used. In our simulations, we have also used this model because we have assumed that even in the environment rich in resources the quantity of nutrients for every cell in a tumor depends on its location within the tumor. The main idea of carrying capacity in logistic models is to set the maximum sustainable population size. This leads to the conclusion that by using only an antiangiogenic inhibitor the vascular network and in turn the tumor can be eradicated.

The second equation describes vascular network growth, including stimulators of angiogenesis (characterized by parameter *γ*), inhibitory factors secreted by tumor cells (*λ*) and natural mortality of the endothelial cells (*μ*). In this model, *N* represents cancer volume,  *β* the proliferation ability of the cells, and *K* the vascular network volume. Inhibitory factors concentrate near the area of the active surface between the tumor and vascular network. The coefficients *ψ*, *η*, *ξ* and are nonnegative constants (conversion factors) that relate the dosages of antiangiogenic (*u*) and cytostatic (*v*) agents as

(1) $$\begin{array}{c} \dot{N}=-\beta N\ln\left(\frac{N}{K}\right)-\psi vN,\\ \dot{K}=\gamma N-\lambda KN^{2/3}-\mu K-\eta uK-\xi vK. \end{array}$$

Based on the Hahnfeldt et al. model, d'Onofrio and Gandolfi proposed some modifications [23]. This model does not take into account the effect of tumor volume relative to the volume of blood on the formation of new blood vessels.

The next modification of these models is the assumption that the increase in vascular network is independent of the size of the tumor, as proposed by Ergun et al. in [24].

In [25], d'Onofrio and Gandolfi analyzed the role of vessel density (which can modulate the effect of drugs) and the effect of vascular “pruning” (by using an antiangiogenic drug in a combined therapy) as

(2) $$\begin{array}{c} \dot{N}=\;-\beta N\ln\left(\frac{N}{K}\right)-\psi \left(\frac{K}{N}\right)vN,\\ \dot{K}=\;\theta \left(h\right)\gamma N-\lambda KN^{2/3}-\;\mu K-\eta uK\;-\xi vK, \end{array}$$

where *h* is the concentration of antiangiogenic agents, exerting a cytostatic action on the endothelial cells, and if there is no such effect, *θ*(*h*) = 1. *K*/*N* is vessel density.

In [26], they proposed included delays in models of process, growth and development of a tumor (*t*_1_) and endothelial cells (*t*_2_). In biological terms, this is the time required for the mitotic division. Delays in the original Hahnfeldt et al. model and d'Onofrio-Gandolfi model were analyzed [27]. The dynamics of the model strongly depends on the place in which the delay is included. In some cases, Hopf bifurcations can occur. Based on this analysis, we calculate the maximal value of delay using parameters proposed in [12]. For *t*_1_ = 0 and *t*_2_ > 0 or *t*_1_ = *t*_2_ > 0, delay could not be greater than 0.2685 and 0.2565, respectively, which is too small to have any effect on protocol dynamics. For *t*_1_ > 0, the maximum value is 12.35, but for small, realistic delays (12 h) there were no significant differences between the results of treatment protocols.

In [13], a new modification was proposed by Benzekry et al. as

(3) $$\begin{array}{c} \dot{N}=-\beta N\ln\left(\frac{N}{M}\right)-\psi vNQM,\\ \dot{M}=\varepsilon I-\tau M,\\ \dot{I}=-\varepsilon I+\gamma N-\lambda IN^{2/3}-\eta uIQM,\\ Q\left(t\right)=\;\frac{M\left(t\right)}{M\left(t\right)+I\left(t\right)}. \end{array}$$

Their idea was based on the original model of Hahnfeldt et al., which includes stable (*M*—mature) and unstable (*I*—immature) vessels. Only stable vessels supply nutrients and oxygen and they are the carrying capacity for cancer cells. Unstable vessels mature with a constant rate denoted by *ε*, and mature vessels have natural mortality *τ*. Stable vessels transport antiangiogenic and cytostatic agents. The quality of the vascular network (*Q*) is calculated and included in factors determining the efficiency of the therapy.

A typical problem observed in chemotherapy is cancer cell resistance to chemotherapy. A three-compartment model was proposed in [28] and includes the Hahnfeldt et al. model of vessel growth and two more equations. The first describes sensitive cancer cells (*S*), and the second resistant cancer cells (*R*). *N*is the sum of all cancer cells as

(4) $$\begin{array}{c} \dot{S}=-aS+\left(1-\upsilon -\frac{S}{K}\right)\left(2-q\right)aS+rcR,\\ \dot{R}=-cR+\left(2-r\right)cR\left(1-\frac{R}{K}\right)+\left(1-\;\upsilon \right)qaS,\\ \dot{K}=\gamma N-\lambda KN^{2/3}-\mu K-\eta uK-\xi vK. \end{array}$$

The coefficients *a* and *c* stand for the inverse of the average transit times through compartments. The probability of mutations occurring during the process is described by *q*, the probability of mutation into the resistive compartment, and *r*, the probability of mutation into the sensitive one. Chemotherapy and antiangiogenic therapy are already incorporated into the equations, with *v* representing the dose of cytostatic killing agent, 0 ≤ *v* ≤ 1 and *u* representing the dose of antiangiogenic drug, and 0 ≤ *u* ≤ 1. As in the original Hahnfeldt model, the coefficients *η*, *ξ*are nonnegative constants (conversion factors) that relate the dosages of antiangiogenic (*u*) and cytostatic (*v*) agents.

A new model for the therapy protocol was proposed by Pinho et al. [29] which is interesting because the equations are not based on the previously discussed ones. The model consists of five differential equations describing successively healthy cells, tumor cells, endothelial cells, cytostatic drug effects, and the impact of antiangiogenic drugs. Additional equations describing therapeutic dynamics are added to the existing ones. 

Another class of models based on ordinary differential equations (three to five) with delays [30] suggest that for rationalizing the empirical results it was necessary to introduce a significant time-delay between the tumor and the vessel formation processes. This might underline the significance of time delays in tumor growth dynamics. Moreover, Hopf bifurcation analysis was performed [30].

Two models that describe tumor growth depending on vascular mass and regulation of new vessel formation through a key angiogenic factor followed by critical-point analysis are presented in [31]. 

A standard Lyapunov-type analysis of stability (local and global) for the Hahnfeldt et al. and d'Onofrio-Gandolfi models was described to find their asymptotic properties [32]. Problems with strongly nonlinear characteristic occur but can be simplified by a logarithmic change of variables and scaling transformations and it is possible to simplify them. A similar analysis was made for Swierniak model [28].

## 3. Optimization of Antiangiogenic Therapy and Combined Therapies

There are many possible strategies in therapy protocol design and testing them all in clinical trials is impossible. Two therapies can be applied at the same time, one after the other or partially overlapping, and one can propose an increasing, decreasing, or constant dose. For this reason, control theory is used to find the best solution.

In [24], the first optimal protocol for antiangiogenic agents combined with radiotherapy for a simple two differential equation models was proposed. Ledzewicz et al. presented a rigorous mathematical treatment of optimal control problem related to antiangiogenic therapy [33]. As a results they obtained optimal strategies containing singular arcs. The same authors obtained a similar optimal strategy containing singular arcs for the original Hahnfeldt et al. model [34]. Different results are obtained for the d'Onofrio-Gandolfi model in the case when TCP (treatment cure probability) under constraints on the cumulative available dose of antiangiogenic agent is optimized for a fixed time of antiangiogenic therapy [35]. The most important conclusion is that intermediate doses of a drug are not optimal and that the optimal protocol contains switches between maximal dose and no drug intervals. Singular arcs are not feasible since there are no finite intervals of constant solutions to the adjoint equations. Similar properties were found for the Hahnfeldt et al. model with logistic tumor growth [32]. Suboptimal strategies for the original Hahnfeldt et al. model for minimization of tumor volume with antiangiogenic therapy using bang-bang optimal controls were described in [36]. The problem to minimize the tumor volume and prevent it from growing using a continuous optimum antiangiogenic drug dose using two controllers was shown in [37]. Simple suboptimal protocols for models with and without a linear pharmacokinetic equation are presented in [38]. The big advantage is that the protocols realize tumor volume dynamics close to the optimal ones. Similar research made by the same group including optimal singular arcs is described in [39]. For piecewise constant dosage protocols, a very good approximation to optimal solutions may be obtained; however, small doses have no significant effect on tumor development, but on the other hand a too high dosage is not efficient enough to justify its enormous cumulative cost.

After the first experimental confirmations of the negative results of single angiogenic inhibitor treatment, preliminary results about optimal controls for a mathematical model that combines antiangiogenic therapy with a chemotherapeutic killing agent were presented [40]. Mathematically, this becomes a multicontrol problem and the structure of a synthesis of optimal controls is significantly more complex than in the monotherapy case. Some optimal strategies for combined antiangiogenic therapy or immunotherapy with chemotherapy were proposed in [41, 42]. The most extensive combination therapy optimization protocols include two cases: combination treatment with angiogenic inhibitors and a cytotoxic agent, and the case when a standard linear pharmacokinetic equation for the antiangiogenic agent is added [38].

In all studies, the most important problem is related to fitting the parameters of the models to the real data. Clinical recommendations based on the results of optimization are possible only in the case when the modeling results can be compared with experimental or clinical trials.

## 4. Results and Discussion

**Figure 1:**
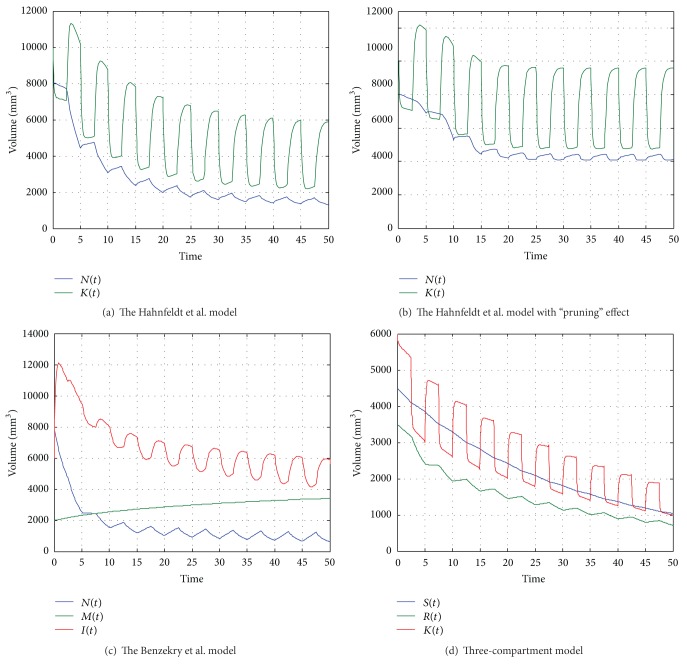
Periodic therapy protocols.

**Figure 2:**
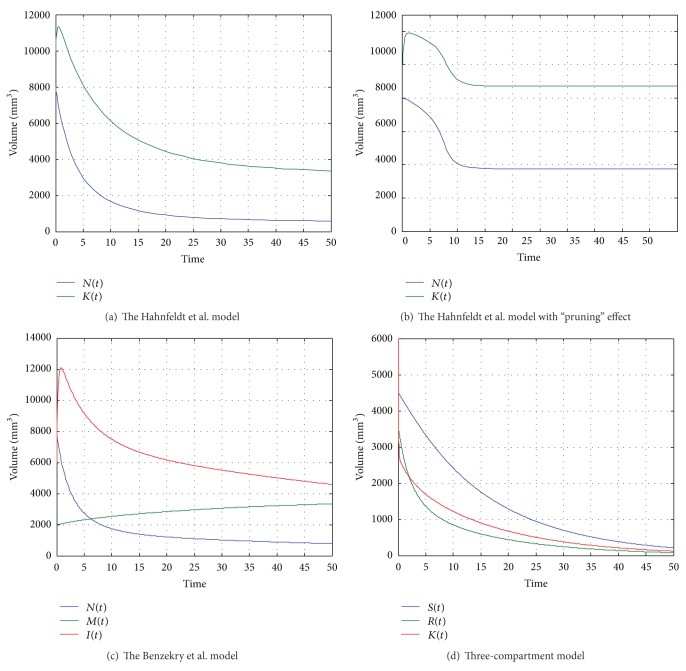
Continuous therapy protocols.

Simple protocols of continuous (Figures 2(a)–2(d)) and periodic (Figures 1(a)–1(d)) therapy were implemented. We used the parameters proposed by Hahnfeldt et al. [12] in order to implement each model under similar conditions. All parameters are summarized in Table 2. In periodic treatment, angiogenic therapy was implemented by first considering that the vascular network should be normalized before chemotherapy. The period for this protocol is 5 days. Detailed results are presented in Table 3 where different doses of antiangiogenic agent and different periods of therapy were examined based on the original Hahnfeldt et al. model. There was no significant variation in tumor volume after therapy when a greater dose was used. In the case of a ten-times lower dose, the effect of therapy was strongly related to the length of the cycle, and for shorter periods the tumor volume was greater than that for longer ones. The dose of antiangiogenic agent is significant in combination with chemotherapy, where the main goal is to improve the structure and function of the tumor vessels. Too aggressive or sustained antiangiogenic treatment may prune away the vascular network, resulting in resistance to further treatment and in difficulties for delivery of drugs or oxygen [43]. This aspect is not included in the original Hahnfeldt et al. model, and for this reason we decided to include the modification of d'Onofrio-Gandolfi [25] suggesting a pruning effect and the division into mature and immature blood vessels suggested by Benzekry et al [13]. We decided to analyze a three-compartment model [28] where resistance to a cytostatic is included, which is one of the most important obstacles against successful cancer cell chemotherapy.

The results of single antiangiogenic therapy for patients and during clinical trials were different. Resistance to angiogenesis inhibitors leads to negative side effects and, in some cases, caused metastatic disease progression. The idea of antiangiogenic therapy is that a tumor cannot grow beyond certain dimensions without developing its own network of blood and lymphatic vessels, while combined with chemotherapy antiangiogenic inhibitors may play an additional function which is normalization of cancer blood vessels.

We describe the comparison of combinations of anticancer therapy protocols for distinct mathematical models. 

During continuous treatment (Figures 2(a)–2(d)), a tumor is easily eliminated, but only when we have assumed that the pruning effect is stabilizing on some level. Based on the function described in [25], we have observed that the best properties of the vascular network are when the ratio (endothelial cells/cancer cells) is 2. If it is larger, the vascular network is unstable, and if smaller there are not enough blood vessels. 

In periodic protocols, the dose of the antiangiogenic agent for Hahnfeldt et al. and its modification from [25] has been increased, due to the fact that the previous value has no effect (the d'Onofrio-Gandolfi modification) or only a small effect (the Hahnfeldt et al. model). The therapeutic effect is smaller than that during the continuous therapy, and the dynamics of all four models is similar. 

These models do not include the factor of hypoxia, which occurs after single antiangiogenesis monotherapy causing proliferation of cancer cell and metastasis. Attention to the importance of this process was paid ten years ago [24]. Examples showed that after antiangiogenic therapy the average survival of patients could be worse than that of patients with no therapy, and that patients receiving a higher dose of antiangiogenic agent had shorter progression-free survival than those receiving a lower dose [10, 44]. Based on the Hahnfeldt et al. model, it is impossible to observe this situation because of the carrying capacity; the tumor is under the control of endothelial cells. This is possible only after including the pruning effect, to find an appropriate function describing the influence of cytostatics and to manipulate the parameters. In the Pinho et al. model [29], the growth is bounded not only by the vascular network but also by its sum with the constant parameter *k*_2_. We have observed that the previous action of the antiangiogenic therapy does not modify the effect of the individual action of the chemotherapy. 

A modification proposed by d'Onofrio-Gandolfi is useful, but implies a new problem: how to describe the influence of cytostatics on cancer cell changes depending on the density of a vessel; a new parameters should also be measured or estimated. A modification proposed recently in [13] was to serve a similar goal; it included mature and immature vessels. However, in this protocol the authors did not include the influence of cytostatics on blood vessels. A new equation was presented, but the dynamics of this model is very similar to the original Hahnfeldt model. The idea of creating mathematical therapy protocols is to find the optimal dose and type of drug for every individual patient, and every factor will be estimated by means of experiment. New biological phenomenon must be described by a minimum of one extraparameter, and consequently more experiments should be performed.

**Figure 3:**
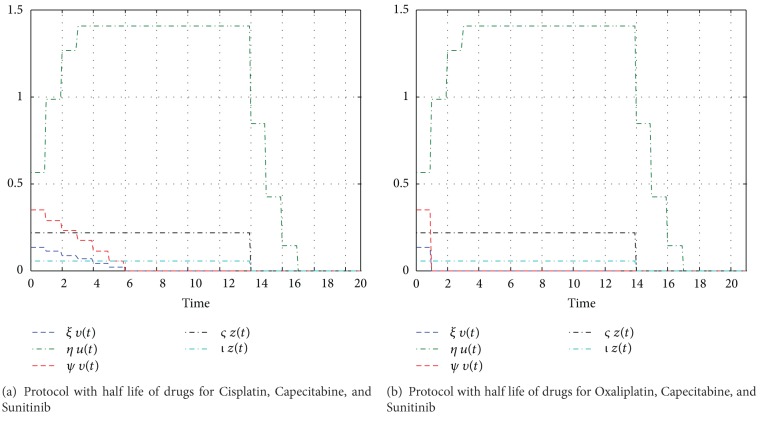
Biological treatment protocols from [11] (NCT00555620).

We investigate the outcome of combined therapy protocols already studied by biologists in which three different drugs are used. Administration of the drugs is as follows: angiogenic inhibitor Sunitinub (SU)—oral capsule daily for 2 weeks (14 days) followed by 1 week (7 days) off treatment; cytostatic drugs—Cisplatin (CIS) (mg/m^2^)—intravenous (IV) on day 1 of each 21-day cycle with Capecitabine (CAP) (mg/m^2^)—oral tablets twice-a-day (BID) on days 1–14 of each 21-day cycle or Oxaliplatin (OXA) (mg/m^2^)—on day 1 of each 21-day cycle with CAP. We assume that 1.7 m^2^ is a standard human surface. The half lives of these agents are Cisplatin 30–100 hours (mean: 65 h ~3 days), Sunitinib 40–60 hours (mean: 50 h ~2 days), Capecitabine 38–45 minutes, and Oxaliplatin ~10–25 minutes. Protocols with included half life are described in Figures 3(a) and 3(b). In the Hahnfeldt et al. model we have included second cytostatic agents which have a direct influence on cancer cells and an indirect influence on endothelial cells (Table 4—parameters). The comparison of results from the mathematical and biological protocols is shown in Table 5. For the protocol including SU, CIS, and CAP, the biological results are quite similar to those obtained by mathematical simulation. The cytostatic drugs have a strong influence for both types of cells, that is why for small doses of antiangiogenic inhibitors and larger cytostatic ones we get better results than for larger doses of angiogenic and smaller doses of cytostatic ones. In the second case, the results for SU, OXA, and CAP are not in agreement with the experimental data. Higher doses of all therapeutics cause a relatively short progression-free survival, in contrast to the mathematical simulation. 

The half-life time of drugs must be taken into account. Cytostatic drugs mostly have a rather short half-life of only a few hours, but the half life of antiangiogenic agents may vary over a wide range, for example, 15 minutes (angiostatin) up to 20 days (bevacizumab).

Resistance to cytostatic agents is one of the most important obstacles against successful cancer cell chemotherapy. Recent tumor research has led scientists to recognize the central role of cancer stem cells (CSCs) in sustaining malignancy and chemoresistance. CSCs have also many intrinsic or acquired properties which seem to be related to tumor drug resistance such as quiescence, specific morphology, DNA repair ability and overexpression of antiapoptotic proteins, drug efflux transporters, and detoxifying enzymes [45].

New therapies acting directly against CSCs are studied by several groups [46]. An interesting model describing the mechanisms that give rise to the different kinds of cancer stem-like cells and the role of these cells in cancer diseases is described in [47]. 

## 5. Conclusions

Many mathematical models of tumor angiogenesis have been proposed, but for analysis and optimization of therapy protocols the most useful seems to be a class of models proposed by Hahnfeldt et al. [12]. After clinical trials, several biological processes have been included as a modification of the original model, related to cytostatic responses, problems with delivery of drugs to tumor cells (because of immature vessels), and delays. These modification, have not changed the dynamics of the models significantly.

Combination of antiangiogenic therapy with conventional treatment is one of the most inspiring approaches in modern oncology. There are also proposals for multiinhibiting formation of tumor blood vessels. From a mathematical point of view, the influence of more than one therapy and not only one kind of drug becomes a multicontrol problem.

The duration of the treatment protocols and cumulated dose of the drugs should be included because of the long half time of some antiangiogenic drugs, their costs, and side effects. 

We have shown that in some cases simple mathematical protocols with varying treatment doses can predict the behavior of tumor growth, but reformulation of models for realistic conditions, including effects of hypoxia which has a significant influence, is required.

## Figures and Tables

**Table 1:** Results from clinical trials of single chemotherapy or combined with an antiangiogenic agent.

ClinicalTrials.gov identifier	Antiangiogenic agents	Cytostatic agents	Progression-free survival
NCT00219557	Axitinib	Gemcitabine Gemcitabine	116 days (109 to 160) 113 days (68 to 205)
NCT00532155	Aflibercept	Docetaxel Docetaxel	5.19 months (4.37 to 5.55) 4.11 months (3.52 to 4.34)
NCT00434252	Bevacizumab	Carboplatin, Paclitaxel Carboplatin, Paclitaxel	5.6 months (4.21 to 6.80) 4.2 months (2.83 to 5.36)
NCT00687297	Vandetanib 4 cycles and maintenance treatment	Docetaxel, Carboplatin	4.5 months (3.3 to 5.8)
	Vandetanib 4 cycles only, no maintenance treatment	Docetaxel, Carboplatin	4.2 months (2.8 to 4.9)

NCT00130728	Erlotinib, Bevacizumab		3.4 months (2.79 to 4.27)
	Bevacizumab		1.7 months (1.48 to 2.53)

**Table 2:** Parameters used in simulation.

Model	Parameter	Description	Value and unit
	*β*	Tumor growth parameter	0.192 day^−1^
	*γ*	Endothelial stimulation parameter	5.85 day^−1^
	*λ*	Endothelial inhibition parameter	0.00873 day^−1^ mm^−2/3^
	*μ*	Natural mortality of endothelial cells	0 day^−1^
Hahnfeldt et al. [12]	*η*	Antiangiogenic killing parameter	0.15 kg mg^−1^
	*ξ*	Cytostatic killing parameter for endothelial cells	0.26 kg mg^−1^
	*ψ*	Cytostatic killing parameter for cancer cells	0.34 kg mg^−1^
	*u*	Dose of angiogenic inhibitor	2 mg kg^−1^ day^−1^
	*v*	Dose of cytotoxic drugs	2 mg kg^−1^ day^−1^

Hahnfeldt et al. with “pruning” effect [25]	Same as in the previous model, *ψ* is different.		
	*ψ*	Cytostatic killing parameter for cancer cells	Depends on vessel density, calculated by equation: $\psi \left(\rho \right)=\frac{\overset{-}{\gamma }}{\left(1+{\left(\left(\rho -\rho_{m}\right)/\sigma \right)}^{2}\right)}$ where, $\overset{-}{\gamma }=0.3$, *ρ*_*m*_ = 2, *σ* = 0.35

	*β*	Tumor growth parameter	0.192 day^−1^
	*γ*	Immature endothelial stimulation parameter	5.85 day^−1^
	*λ*	Immature endothelial inhibition parameter	0.00873 day^−1^ mm^−2/3^
	*ε*	Unstable vessels maturation parameter	0.0756 day^−1^
Benzekry et al. [13]	*τ*	Natural mortality of mature endothelial cells	0.075 day^−1^
	*η*	Antiangiogenic killing parameter	6.85 × 10^−7^ mg^−1^ mm^−1^
	*ψ*	Cytostatic killing parameter for cancer cells	1.37 × 10^−5^ mg^−1^ mm^−1^
	*u*	Dose of angiogenic inhibitor	525 mg day^−1^ (half dose during continuous treatment)
	*v*	Dose of cytotoxic drugs	212 mg week^−1^ (half dose during continuous treatment)

Three-compartment [28]	*a*	Average transit times through compartments	0.02 day
	*c*	Average transit times through compartments	0.2 day
	*q*	Probability of mutation to resistant cell	0.9
	*r*	Probability of mutation to sensitive cell	0
	*γ*	Endothelial stimulation parameter	5.85 day^−1^
	*λ*	Endothelial inhibition parameter	0.00873 day^−1^ mm^−2/3^
	*η*	Antiangiogenic killing parameter	9.1 kg mg^−1^
	*ξ*	Cytostatic killing parameter for endothelial cells	4.7 kg mg^−1^
	*u*	Dose of angiogenic inhibitor	1 mg kg^−1^ day^−1^
	*v*	Dose of cytotoxic drug	1 mg kg^−1^ day^−1^

**Table 3:** Results of anti-angiogenic therapy combined with chemotherapy for different periods and dosage.

Period	Total time of treatment	Tumor value after periodic treatment with dose of cytostatics inhibitors 1 mg kg^−1^ day^−1^	
		RowSpanEmpty	Anti-angiogenic agents dose 2 mg kg^−1^ day^−1^	Anti-angiogenic agents dose 20 mg kg^−1^ day^−1^
2 days	50 days	3525.1 mm^3^	1179.8 mm^3^
3 days	48 days	3402.4 mm^3^	1265.1 mm^3^
4 days	48 days	3267.5 mm^3^	1306.9 mm^3^
5 days	50 days	3121.1 mm^3^	1315.0 mm^3^
6 days	48 days	3019.0 mm^3^	1337.2 mm^3^
7 days	49 days	2891.9 mm^3^	1334.3 mm^3^
8 days	48 days	2781.7 mm^3^	1338.4 mm^3^
9 days	45 days	2689.0 mm^3^	1351.5 mm^3^
10 days	50 days	2576.8 mm^3^	1312.2 mm^3^
11 days	44 days	2492.2 mm^3^	1339.6 mm^3^
12 days	48 days	2400.3 mm^3^	1301.4 mm^3^
13 days	39 days	2333.0 mm^3^	1360.0 mm^3^
14 days	42 days	2241.6 mm^3^	1318.6 mm^3^

**Table 4:** Proposed parameters.

Model	Parameter	Description	Value and unit
	*β*	Tumor growth parameter	0.192 day^−1^
	*γ*	Endothelial stimulation parameter	5.85 day^−1^
	*λ*	Endothelial inhibition parameter	0.00873 day^−1^ mm^−2/3^
	*μ*	Natural mortality of endothelial cells	0 day^−1^
	*η*	Anti-angiogenic killing parameter	0.01 mg^−1^
	*ξ*	Cytostatic killing parameter Cisplatin (for endothelial cells)	0.0013 m^2^ mg^−1^
Hahnfeldt et al. [12]	*ψ*	Cytostatic killing parameter Cisplatin (for cancer cells)	0.004 m^2^ mg^−1^
	*ξ*	Cytostatic killing parameter Oxaliplatin (for endothelial cells)	0.005 m^2^ mg^−1^
	*ψ*	Cytostatic killing parameter Oxaliplatin (for cancer cells)	0.01 m^2^ mg^−1^
	*σ*	Cytostatic killing parameter Capecitabine (for cancer cells)	0.00008 m^2^ mg^−1^
	*ι*	Cytostatic killing parameter Capecitabine (for endothelial cells)	0.00002 m^2^ mg^−1^
	*u*	Dose of angiogenic inhibitor	Depends on protocol
	*v*	Dose of cytotoxic drug Cisplatin	
	*z*	Dose of cytotoxic drug Capecitabine	

**Table 5:** Comparison of results from mathematical and biological protocols.

Protocol	Progression-free survival	Tumor volume after 21 days simulation
SU 37.5 mg CIS 60 mg/m^2^, CAP 1600 mg/m^2^	3.2 months (2.7 to 9.3)	5249.1 mm^3^
SU 37.5 mg CIS 60 mg/m^2^, CAP 2000 mg/m^2^	6.6 months (2.5 to 8.0)	4071.5 mm^3^
SU 25 mg CIS 80 mg/m^2^, CAP 2000 mg/m^2^	6.4 months (4.3 to 13.9)	4370.3 mm^3^
SU 37.5 mg OXA 110 mg/m^2^, CAP 1600 mg/m^2^	8.0 months (4.7 to 9.4)	4963.9 mm^3^
SU 37.5 mg OXA 110 mg/m^2^, CAP 2000 mg/m^2^	2.8 months (2.3 to 11.7)	3830.0 mm^3^
SU 25 mg OXA 110 mg/m^2^, CAP 2000 mg/m^2^	5.5 months (4.7 to 10.1)	4623.9 mm^3^
